# Clinical application of drug sensitive gene detection in postoperative instillation for non-muscle invasive bladder cancer

**DOI:** 10.1186/s12882-020-02073-4

**Published:** 2020-10-07

**Authors:** Zhenlong Wang, Hui Tang, Yuquan Xue, Li Xue, Hongliang Li, Tie Chong

**Affiliations:** Department of Urology, The Second Affiliated Hospital of Xi’an Jiaotong University, No.157 Xiwu Road, Xi’an, 710004 Shaanxi China

**Keywords:** Non-muscle invasive bladder cancer, Pharmacogenomic testing, Chemotherapy drugs, Bladder instillation

## Abstract

**Background:**

Bladder cancer is the most common malignant tumor of the urinary system. One of the biological characteristics of NMIBC is the high recurrence rate after surgery. The implementation of this project aimed to investigate the role of pharmacogenomic testing-guided intravesical perfusion of chemotherapeutic agents in the postoperative perfusion therapy for non-muscle invasive bladder cancer.

**Method:**

From January 2015 to December 2016, 298 patients with non-muscle-invasive bladder cancer were enrolled in this prospective study. These patients received chemotherapy drugs after electrotherapy. According to the presence or absence of tumor susceptibility gene detection after surgery, they were divided into two groups, including the drug sensitive group(*N* = 44) and the control group(*N* = 254). The drug sensitive group received bladder infusion therapy with sensitive chemotherapy drugs based on drug sensitivity gene detection results. The control group received intravesical instillation of pirarubicin. The preoperative general data and tumor grade of patients were recorded. Cystoscopy was performed before and every 3 months after surgery. The chest CT, upper abdomen CT, renal function, and urinary routine tests were performed. Tumor recurrence, metastasis and tumor-related death were recorded and evaluated during follow-up.

**Results:**

The drug sensitive group, which selected high-sensitivity drugs for intravesical instillation therapy based on gene expression, has a significantly lower relapse rate (11.36% vs 37.40%, *P* < 0.05) and a significantly longer time to relapse **(**17.80 ± 7.20 month vs11.20 ± 6.10 month, *P* < 0.05) compared with the control group. There were no significant differences in the time of mortality and death time between two groups.

**Conclusion:**

The pharmacogenomic testing-directed bladder instillation of chemotherapeutic drugs may be more effective than empiric drug administration in reducing the recurrence rate of non-muscle-invasive bladder cancer.

## Background

Bladder cancer is the most common malignant tumor of the urinary system [1], and non-muscle invasive bladder cancer (NMIBC) accounts for 75–80% of the total incidence. One of the biological characteristics of NMIBC is the high recurrence rate after surgery. Intravesical instillation of chemotherapy drugs for NMIBC after transurethral resection of bladder tumor (TURBT) is the main method to reduce the rate of postoperative recurrence. However, it is been reported that the recurrence of tumor still occured in 15–61% of patients during treatment [2]. Drug resistance is one of the main factors leading to tumor recurrence, which has become a major obstacle for the treatment of bladder cancer. To prevent drug resistance [3], drug sensitivity prediction may contribute to improve the efficacy of local infusion chemotherapy for bladder cancer.

At present, there are many kinds of chemotherapeutic drugs for intravesical instillation. However, there is no recognized drug with absolute therapeutic advantages. The drug selection is mostly based on empirical drug use. There is no uniform rational application guideline for these drugs, leading to some patients being resistant to these chemotherapeutic drugs. However, there are few reports on the individualized treatment of bladder cancer through the guidance of drug gene detection. The implementation of this project aimed to develop a plan for bladder infusion medication for clinicians, to achieve standardized and individualized treatment of bladder perfusion after bladder tumor surgery, further to reduce the recurrence rate of tumor after surgery. It has practical significance and clinical application value.

## Methods

### General information and grouping of patients

In this prospective study, patients who underwent elective bladder tumor resection in NMIBC admitted to our department from January 2015 to December 2016 were enrolled and followed up for more than 1 year. According to the presence or absence of drug sensitivity gene detection, patients were divided into 2 groups: 1) Drug-sensitive group: After TURBT, based on the detection of tumor tissue gene markers, sensitive chemotherapy drugs were selected for postoperative bladder infusion therapy. 2) Control group: After TURBt, gemcitabine, pirarubicin, and mitomycin were used for bladder infusion according to the surgeon’s experience. This study was reviewed and approved by the Medical Ethical Committee of the The Second Affiliated Hospital of Xi’an Jiaotong University. Written informed consent was obtained from all subjects.

The inclusion criteria were as follows: according to the 2016 AUA NMIBC risk classification [4], medium and low risk NMIBC patients,1) patients who at any age with any gender from any region were suggestive of urothelial cells NMIBC by preoperative imaging examination and postoperative pathology; 2) who underwent urethral bladder tumor resection and received intravesical instillation with postoperative gemcitabine, pirarubicin/epirubicin or mitomycin; 3) who didn’t receive any systemic treatment, local chemotherapy or immunotherapy that will affect the final outcome before and during the study; 4) the total follow-up time was greater than 1 year; 5) who had detailed report on the outcome.

The exclusion criteria were as follows: 1)According to the 2016 AUA NMIBC risk grading high-risk patients and patients with myometrial invasive bladder cancer; 2) patients did not complete bladder infusion for any reason; patients discontinued perfusion therapy due to adverse reactions occurred during bladder chemotherapy drug infusion and are still intolerant after symptomatic treatment; 3) patients combined with severe cardiorespiratory multi-system diseases that affect perfusion therapy, such as liver and kidney dysfunction, and electrolyte imbalance; 4) postoperative pathology confirmed non-urothelial cell carcinoma, such as bladder squamous cell carcinoma and adenocarcinoma; 5) failed to obtain contact after surgery and follow-up loss.

### Drug sensitivity gene detection method

#### Drug sensitive gene selection

By searching a large number of literatures, we have selected several confirmed and well-recognized drug gene markers RRM1, TOP2A, BCL-2 mRNA that are sensitive to the commonly used bladder infusion drugs, including gemcitabine, pirarubicin/epiubicin and mitomycin.

#### Sample collection

In the drug-sensitive group, 2 specimens of tumor tissue were taken after TURBT. One specimen was used for the detection of the drug-sensitive gene markers, the other was used for pathological examination. Tumor tissue samples originated from the control group were used for pathological examination.

#### Detection of drug-sensitive gene markers in drug-sensitive group

The multiplex branched-DNA (bDNA) liquidchip technology was applied in this study [5, 6], signal amplification was achieved by lysing samples, microsphere capture, probe multi-site-specific pairing, cascade amplification, and quantitative detection of the mRNA expression of target genes, including RRM1, TOP2A and BCL-2. The liquid crystal chip was used for the interpretation of the experimental results [7].

#### Detailed experimental steps

1. Sample lysis: took appropriate amount of formalin-fixed paraffin-embedded (FFPE) sample and added lysis buffer, incubated at 56 °C for 2 h;
2. Pre-hybridization: transfered the sample lysate to the incubation plate, added support probe-microsphere, support extension probe and buffer, shaking at 55 °C overnight;
3. Supernatant aspiration: the incubation plate was placed on a magnetic stand for 1 min, the magnetic microspheres at the bottom were collected, and the supernatant was removed by aspiration;
4. Washing: added washing solution, shaking and washing for 1 min. The incubation plate was placed on a magnetic stand for 1 min, and the supernatant was discarded by aspiration; this step was repeated 3 times;
5. Hybridization: addition of an amplification probe and a labeled probe, shaking at 50 °C for 1 h; 6) Washing: incubating plate was placed on a magnetic stand for 1 min, aspirated and discarded the supernatant. Then, the plate was washed twice with washing solution;
6. Signal amplification: added streptavidin-phycoerythrin, shaking at 50 °C for 30 min;
7. Washing: incubating plate was placed on magnetic stand for 1 min, aspirated and discarded the supernatant. Then, the plate was washed twice with washing solution;
8. Reading: added washing solution, shaking for 5 min, and the data were read by Luminex reader;
9. Data collection and analysis: the raw results could be transformed into the microsphere median fluorescence reading value by the software, which provided data processing to produce the final result.

#### Interpretation of drug sensitivity test results

The levels of gene expression in tumor tissue are classified as following 5 grades: more than 75%, 60–75%, 40–60%, 25–40% and less than 25%. The efficacy of gemcitabine is negatively correlated with the expression level of RRM1 gene, indicating the low expression of RRM1 gene indicates sensitive to gemcitabine. The curative effect of anthracyclines (including piriubicin and epirubicin) is positively correlated with the expression level of TOP2A gene, indicating the high expression of TOP2A gene is sensitive to anthracyclines. Meanwhile, mitomycin C sensitivity is negatively correlated with Bcl-2 gene expression, and thus low expression of Bcl-2 gene is sensitive to mitomycin C [8–10] .

### Bladder perfusion and follow-up of patients

The dosages of chemotherapy drugs were as follows: pirarubicin 30 mg/time, gemcitabine 1000 mg/time, mitomycin 20 mg/time. After TURBT, these chemotherapy drugs were applied in the process of immediate perfusion within 6 h. Bladder perfusion was performed once a week, perfused for 8 weeks, and then reperfused once a month for 10 months. Intravesical instillation treatment time was 1 year. In the drug-sensitive group, gemcitabine, pirarubicin or mitomycin was selected for each patient based on the drug sensitivity test results; while these drugs were used empirically by the competent physician in the control group.

After surgery, postoperative follow-up plan was carried out for all the patients every 3 months for 2 years, and they were followed up for at least 1 year. The Follow-up items included chest CT, upper abdomen CT, renal function, urine routine test and cystoscopy. Tumor recurrence, metastasis and tumor-related death were recorded and evaluated during follow-up.

### Statistical analysis

All the data collected in this study were analyzed using SPSS 22.0 software. Measurement data were expressed as mean ± standard deviation (mean ± SD), and the comparisons of two groups were examined by t-test. The categorical data were expressed as n(%), and their differences between two groups was examined by chi-squared test. P<0.05 was considered statistically significant.

## Results

### General data of the two groups of patients

From January 2015 to December 2016, 298 patients with non-muscle invasive bladder cancer who underwent chemotherapy after perfusion of bladder tumors were enrolled in our study, including 44 patients in the drug-sensitive group and 254 patients in the control group. The average age was 63.40 ± 12.30 years old. According to 2004 WHO non-invasive urothelial carcinoma pathological classification, 102 cases were classified as low-grade malignant potential urothelial papilloma, 138 cases were low-grade urothelial carcinoma, and 58 cases were high-grade urothelial carcinoma. There were 256 cases of single tumor and 42 cases of multiple tumors. There were no significant differences in gender, age, tumor grade, predilection location and number of tumors between the drug-sensitive group and the control group (*P* > 0.05) (Table 1).

**Table 1:** General data of patients

	Drug sensitive group (N = 44)	Control group (N = 254)	*P* value	χ^2^ value
Gender (male/female)	32/12	203/51	0.281	1.164
Age (years old)	62.6 ± 14.30	63.8 ± 12.05	0.076	1.635
Tumor grade			0.556	1.173
Low-grade malignant potential urothelial papilloma	25	84		
Low grade urothelial carcinoma	15	121		
High-grade urothelial carcinoma	4	49		
Tumor number			0.189	1.725
Single	35	217		
Multiple	9	37		
Tumor location			0.763	1.759
Front wall	6	43		
Back wall	14	96		
Side wall	17	89		
Triangle area and bladder neck	7	37		

### Drug sensitivity gene detection and chemotherapeutic drug selection results

Based on the gene expression results (Table 2), gemcitabine was selected for treatment of the patients with low expression of RRM1 gene. For patients with high expression of TOP2A gene and low expression of Bcl-2 gene, pirarubicin and mitomycin were selected for the bladder perfusion, respectively. As a result, the application of gemcitabine, pirarubicin and mitomycin in the drug-sensitive group for bladder perfusion were 38.63, 43.18 and 18.18%, respectively; while the application of gemcitabine, pirarubicin and mitomycin in the control group for bladder perfusion were 35.43, 41.73 and 22.83%, respectively. There was no significant difference in the application of these drugs between two groups (P>0.05) (Table 3).

**Table 2:** Drug sensitivity gene expression

Expression level	RRM1	TOP2A	BCL-2
more than 75%	10	15	3
60–75%	3	6	4
40–60%	8	3	27
25–40%	6	7	2
less than 25%	17	13	8

**Table 3:** Bladder perfusion drug selection distribution

	Gemcitabine	Pirarubicin	Mitomycin
Drug sensitive group	17 (38.63%)	19 (43.18%)	8 (18.18%)
Control group	90 (35.43%)	106 (41.73%)	58 (22.83%)
χ^2^ value		0.658	
*P* value		0.065	

### Comparison of bladder perfusion effectiveness in drug-sensitive group

Compared with the control group, the recurrence rate of bladder tumor in the drug-sensitive group was significantly lower (*P* < 0.05). There were no significant differences in the rate of metastasis, metastasis time, mortality and death time between two groups (P>0.05) (Table 4).

**Table 4:** Comparison of prognosis between drug-sensitive group and control group

		Drug sensitive group (N = 44)	Control group (N = 254)	χ^2^/t	*P* value
Relapse	Rate	11.36%(5/44)	37.40%(95/254)	11.404	0.010
Metastasis	Rate	4.54%(2/44)	3.14%(8/254)	0.225	0.635
	Time (month)	20.00 ± 2.83	27.85 ± 12.10	2.263	0.406
Death	Rate	6.81%(3/44)	7.48%(19/254)	1.265	0.809
	Time (month)	22.00 ± 3.46	23.33 ± 9.01	0.024	0.877

### Comparison of tolerance and safety between the two groups

Some adverse evens occurred during perfusion of bladder chemotherapy drugs, mainly manifested as varying degrees of bladder irritation. Most occurred in the bladder perfusion for 4–6 weeks. There was no significant difference in the incidence of bladder irritation between the two groups (88.64% vs 89.76%, *P* > 0.05). Among them, 32 patients had bladder irritation. The M receptor blockers and phytopharmaceuticals were used in treatment of mild patients, and those moderate to severe patients can be given bladder mucosal protective agent (e.g. sodium hyaluronate) for bladder infusion.

## Discussion

The liquid phase chip technique detects the mRNA expression level and gene mutation of the sample [11]. Direct detection of mRNA expression can be achieved without RNA extraction, purification and reverse transcription. The detection results are basically not affected by factors such as RNA degradation in the sample, which ensures the accuracy of the detection results; on the other hand, multiple housekeeping genes are used as controls, so the test results are not affected by pathological conditions, thereby improving the reliability of the test results. At the same time, the liquid phase chip uses a multi-site-specific pairing and cascade amplification of the probe to realize signal amplification instead of PCR amplification method, which improves the detection signal and achieves the specificity of detection and avoids the false positive of reverse transcription PCR and real-time fluorescent quantitative PCR technology. The high-throughput of liquid phase chip technology and the high accuracy of the detection system enable the simultaneously detection of the mRNA expression level of multiple target genes in one reaction. Thus, the liquid phase chip technique can avoid the detection difference caused by different mRNA loading, and ensure the sensitivity and specificity of the detection.

Bladder cancer is one of the most common malignant tumors in the genitourinary system. In recent years, the incidence rate has increased year by year. Related reports have shown that in 2015, the number of patients with bladder cancer in China was approximately 80,500, and the number of deaths was about 32,900 [12] 90% of bladder tumors are urothelial carcinomas, which have the characteristics of multi-center, easy recurrence, drug resistance and easy invasion. Some recurrent tumors with increased malignancy may progress to invasive or metastatic cancer [13] According to the graded stage of the tumor and the patient’s own situation, a variety of treatments for bladder cancer can be selected, including transurethral resection of bladder tumor, transurethral resection of bladder tumor, partial resection of the bladder, radical resection of the bladder, and so on. Although surgical treatment has been gradually popularized in the clinic, the effect of non-surgical treatment such as bladder cancer infusion chemotherapy, systemic chemotherapy, radiotherapy is still not ideal [14]. Nevertheless,there is still no recognized and universally applicable bladder cancer diagnosis and prognosis evaluation markers. At present, intravesical instillation of transurethral non-muscle invasive bladder tumor has been recognized as one of the conventional treatments that can effectively reduce the recurrence rate of patients with superficial bladder cancer [15].

The anti-tumor mechanism of various bladder infusion drugs is different, and the effects and related side effects are different. For low- and medium-risk NMIBC, more literature has confirmed that chemotherapy drugs are the preferred bladder infusion drugs. There are many kinds of chemotherapy drugs for bladder infusion, including mitomycin MMC, epirubicin EPI, and gemcitabine. However, there is currently no recognized drug with absolute therapeutic advantages. There is no uniform guide to the rational use of drugs. Most drug choices are empirical drugs, which cause some patients to be insensitive to the drug, so that 15–61% of patients still have tumor recurrence after surgery [2]. The results of this study showed that the rate of tumor recurrence after surgical selection of NMIBC patients reached 37.40%. Therefore, how to choose sensitive chemotherapy drugs, guide individualized bladder perfusion, and achieve standardized application of chemotherapy drugs in local treatment of bladder cancer is the key to reduce postoperative tumor recurrence.

Chemotherapy sensitivity detection of tumors is a requirement for standardized application of chemotherapy drugs, and is a trend of individualized treatment of tumors. Therefore, how to choose sensitive chemotherapy drugs, guide individualized bladder perfusion, and achieve standardized application of chemotherapy drugs in local treatment of bladder cancer is the key to reduce postoperative tumor recurrence. At present, in the adjuvant chemotherapy of lung cancer and liver cancer, drug-related molecular markers can be used to predict drug sensitivity and drug resistance, and the risk of toxicity, and then guide the individualized and standardized use of chemotherapy drugs. Clinical studies have focused on the evaluation of drug sensitivity in advanced bladder cancer for systemic chemotherapy. The results of the existing literature indicate that platinum drugs and mitomycin sensitivity are associated with tumor ERCC1 expression [7], gemcitabine sensitivity and negative RRM1 expression. Correlation [8], anti-microtubule drug sensitivity and TUBB3 expression, anthracycline sensitivity and TOP2A expression are positively correlated [9]. Whether the above research conclusions are equally applicable to the application of chemotherapy drugs to intravesical local perfusion therapy can be used as the basis for our selection of sensitive bladder infusion drugs. Therefore, it is of great value to further study the drug sensitivity test before chemotherapy infusion of chemotherapeutic drugs, and then guide drug selection.

The corresponding molecular markers confirmed by the literature for predicting the sensitivity of chemotherapeutic drugs do not change due to cancerous species. Therefore, the results of susceptibility gene studies of other tumors can also be applied to the detection of bladder tumors and to guide the selection of bladder perfusion sensitive drugs. In this topic, three commonly used bladder infusion chemotherapy drugs, gemcitabine, pirarubicin, and mitomycin, were selected. According to the results of susceptibility genomics literature, patients with low expression of RRM1 gene were treated with intraperitoneal infusion of gemcitabine, pirarubicin was selected for high expression of TOP2A gene, and mitomycin was selected for low expression of Bcl-2 gene [10]. When it is met, choose a more relevant drug.

In this study, the drug sensitivity group according to the drug sensitivity test results, the drug application distribution was as follows: gemcitabine use rate was 38.63%; pirarubicin use rate was 43.18%; mitomycin use rate was 18.18%. The intravesical local recurrence rate of the drug-sensitive group was 11.36%, which was significantly lower than that of the control group (37.40%, *P* < 0.05). The reason is that, compared with the empirical selection of bladder infusion drugs, bladder perfusion drug selection guided by the drug-sensitive gene detection results of bladder tumor tissue can prevent bladder cancer patients from recurrence due to drug resistance. Therefore, bladder infusion guided by drug-sensitive gene detection can significantly reduce the recurrence rate of bladder tumors after NMIBC.

This study also found that there was a significant difference in the recurrence rate between the two groups, but there was no difference between the metastasis rate and the mortality rate. Kurth et al. [16] considered that there are three main factors in the metastasis of non-muscle invasive bladder cancer, namely tumor size, histological grade and whether it is initial. Millan-Rodriguez et al. [17] study that the main factors of NMIBC metastasis are the number of tumors, tumor diameter greater than 3 cm and carcinoma in situ. Kiemeney et al. [18] in multiple cases (1674 cases) showed that tumor staging is an important predictor of bladder cancer metastasis and even death. Bladder tumor staging can understand the size and number of tumors, lymph node involvement, depth of invasion and the presence of metastatic lesions. Closely related to recurrence and metastasis. Shen et al. [19] scholars suggest that the higher the tumor stage, the easier it is to relapse and metastasize. Because of the data, tumor single and multiple, tumor site, and tumor stage, there was no significant difference between the control group and the drug sensitivity group (*P* > 0.05), so the postoperative NMIBC metastasis rate and mortality in this study. There was no statistical difference (*P* > 0.05). Both the study and the above literature suggest that bladder perfusion is effective in reducing the recurrence rate, but it does not reduce the mortality and metastasis rate of patients.

## Conclusion

In conclusion, according to the detection of drug-sensitive genes in bladder tumor tissue, the selection of bladder infusion drugs for superficial bladder cancer can significantly reduce the recurrence rate of bladder tumor after NMIBC. Provide guidance for bladder infusion drug selection after NMIBC. It provides a clinical basis for the standardized individualized treatment of bladder infusion chemotherapy after bladder tumor surgery.

## Data Availability

The datasets used and/or analyzed during the current study available from the corresponding author on reasonable request.

## Abbreviations

FFPE: Formalin-fixed paraffin-embedded
NMIBC: Non-muscle invasive bladder cancer
TURBT: Transurethral resection of bladder tumor
